# Clinical impact of glucocorticoid responsiveness-related gene polymorphism on graft-versus-host disease and survival after single-unit cord blood transplantation

**DOI:** 10.1007/s12185-025-04112-y

**Published:** 2025-11-20

**Authors:** Takaaki Konuma, Megumi Hamatani-Asakura, Maki Monna-Oiwa, Seiko Kato, Shohei Andoh, Hirona Ichimura, Kazuaki Yokoyama, Yasuhito Nannya, Satoshi Takahashi

**Affiliations:** 1 Department of Hematology/Oncology, The Institute of Medical Science, The University of Tokyo, 4-6-1, Shirokanedai, Minato-Ku, Tokyo, 108-8639 Japan; 2 Division of Clinical Precision Research Platform, The Institute of Medical Science, The University of Tokyo, Tokyo, Japan

**Keywords:** Glucocorticoid-induced transcript 1, Nuclear receptor subfamily 3 Group C member 1, Glucocorticoid sensitivity, Graft-versus-host disease, Cord blood transplantation

## Abstract

Graft-versus-host disease (GVHD) is a major complication after allogeneic hematopoietic cell transplantation (HCT), and glucocorticoids remain the standard first-line treatment. However, glucocorticoid responses vary widely and are influenced by genetic polymorphisms in glucocorticoid signaling pathways. Their impact after cord blood transplantation (CBT) is unclear. We retrospectively analyzed adult patients who underwent single-unit CBT at our institution between 2005 and 2023 with available donor or recipient DNA. Genotyping of *NR3C1* (rs33388) and *GLCCI1* (rs37972, rs37973) was performed using TaqMan® assays. In univariate analyses, donor *GLCCI1* rs37973 AG/AA genotypes were associated with a higher incidence of grades II to IV acute GVHD but a lower incidence of chronic GVHD compared with GG donors. Multivariate analysis confirmed that *GLCCI1* rs37973 AG/AA donors had a significantly reduced risk of chronic GVHD (hazard ratio 0.57, 95% confidence interval 0.35–0.93, *P* = 0.025). Among 30 patients who developed grades III to IV acute GVHD treated with glucocorticoids, recipient *NR3C1* rs33388 TT genotype was associated with significantly lower overall survival compared with AT/AA (24.4% vs. 63.5% at 5 years, *P* = 0.044). These findings suggest that donor *GLCCI1* rs37973 and recipient *NR3C1* rs33388 polymorphisms may influence GVHD risk and survival after CBT.

**Supplementary Information:**

The online version contains supplementary material available at 10.1007/s12185-025-04112-y.

## Introduction

Graft-versus-host disease (GVHD) is one of the major complications after allogeneic hematopoietic cell transplantation (HCT). First-line treatment for acute GVHD is based on glucocorticoids. Approximately half of those patients achieved a complete or partial response, but the remaining patients do not respond to initial treatment of glucocorticoids, which could result in poor prognosis after allogeneic HCT [1]. The way the body responds to glucocorticoids in cases of steroid-resistant GVHD is influenced by complicated interactions at the molecular, cellular and tissue levels [2]. Previous studies have shown that how well a case responds to glucocorticoids, or why they might not respond, is linked to differences in gene polymorphism associated with glucocorticoid signaling, like the glucocorticoid receptor and *glucocorticoid-induced transcript 1 (GLCCI1)*, in patients with pemphigus vulgaris, asthma, and acute lymphoblastic leukemia [3–5].

Cord blood transplantation (CBT) is a viable therapeutic alternative for patients lacking a related or unrelated donor. In contrast to allogeneic HCT from other donors, the lower incidence of GVHD is notable in CBT [6–8]. Several studies have shown the associations between polymorphism of genes involving glucocorticoid responsiveness and allogeneic HCT outcomes from other donors [9–11], but the impacts of polymorphisms involving glucocorticoid responsiveness on CBT outcomes have been unclear. Here, we investigated the effect of recipient and donor glucocorticoid receptor, which is encoded by the *nuclear receptor subfamily 3, Group C, member 1 (NR3C1)*, and *GLCCI1* polymorphisms on outcomes in adults who underwent CBT at our institution.

## Methods

### Study design

This retrospective study included adult patients who received single-unit CBT at our institution between January 2005 and March 2023 and for whom recipient or donor DNA samples were available. The Institutional Review Board of the Institute of Medical Science, the University of Tokyo approved this retrospective study (26–112–270,402, 2020–1–0422).

### Genotyping of NR3C1 and GLCCI1

The Gentra Puregene® Blood Kit (Qiagen, Hilden, Germany) was utilized to extract genomic DNA from whole blood or a buccal swab. Genotyping of *NR3C1* (rs33388) and *GLCCI1* (rs37972 and rs37973) was conducted via real-time polymerase chain reaction utilizing the Bio-Rad CFX Connect Real-Time System® (Bio-Rad, CA, USA) with TaqMan® SNP genotyping assays (Thermo Fisher Scientific, MA, USA) and TaqPathTM ProAmpTM Master Mix® (Thermo Fisher Scientific).

### Definitions

The diagnosis and severity of acute GVHD were determined using previously established standard criteria [12]. The definition and classification of chronic GVHD followed the criteria set by the National Institutes of Health (NIH) [13]. The pre-engraftment syndrome (PES) or pre-engraftment immune reaction (PIR) was defined noninfectious fever > 38.3 °C with an unexplained erythematous skin rash occurring before or at neutrophil engraftment [14]. Bacterial bloodstream infection (BSI) was defined as the isolation of bacteria from blood cultures obtained from the day of CBT through day 100 after CBT. Cytomegalovirus (CMV) reactivation was defined as the first detection of CMV pp65 antigenemia (C10/C11) from the time of neutrophil recovery up to 100 days after CBT. Relapse was defined as evidence of underlying disease. Non-relapse mortality (NRM) was defined as death without disease relapse or progression. Overall survival (OS) was defined as the time from CBT to death or the date of last contact. The HCT-specific comorbidity index (HCT-CI) [15] and the refined Disease Risk Index (DRI) [16] were classified according to published criteria. The number of HLA disparities was defined as a low resolution for HLA-A, -B, and -DR in the graft-versus-host direction.

### Statistical analysis

The evaluations of GVHD, PES/PIR, BSI, CMV reactivation, relapse, and NRM were performed utilizing the cumulative incidence method, considering competing risks, and Gray’s test. OS was evaluated utilizing the Kaplan–Meier method and log-rank test. In multivariate analyses, a Fine and Gray model was employed for the endpoints with competing risks, whereas a Cox’s proportional hazards model was utilized for OS. Multivariate analyses incorporated the following covariates: recipient age (< 50 years vs. ≥ 50 years), HCT-CI (0–2 vs. ≥ 3), refined DRI (low/intermediate vs. high/very high), cryopreserved cord blood CD34^+^ cell dose (< 1.0 × 10^5^/kg vs. ≥ 1.0 × 10^5^/kg), number of HLA disparities (0–1 vs. 2), sex incompatibility (female donor to male recipient vs. others), and GVHD prophylaxis (cyclosporine and methotrexate vs. others), along with recipient or donor rs33388 (TT vs. AT or AA), rs32792 (CC vs. TC or TT), or rs37973 (GG vs. AG or AA). All statistical analyses were performed using EZR version 1.68 (Saitama Medical Center, Jichi Medical University, Saitama, Japan) [17]. Statistical significance was defined using a two-tailed *P*-value < 0.05.

## Results

### Patient characteristics

Patient and CBT characteristics are shown in Table 1. Among recipients, the rs33388 genotypes were TT in 65.8% (*n* = 104), AT in 31.7% (*n* = 50), and AA in 2.5% (*n* = 4); among donors, frequencies were TT in 67.7% (*n* = 92), AT in 30.2% (*n* = 41), and AA in 2.2% (*n* = 3). For rs37972, recipients were CC in 37.3% (*n* = 59), TC in 15.2% (*n* = 24), and TT in 47.5% (*n* = 75); donors were CC in 34.6% (*n* = 47), TC in 19.9% (*n* = 27), and TT in 45.6% (*n* = 62). Among recipients, rs37973 genotypes were GG in 17.1% (*n* = 27), GA in 52.5% (*n* = 83), and AA in 30.4% (*n* = 48); among donors, genotypes were GG in 25.7% (*n* = 35), GA in 47.1% (*n* = 64), and AA in 27.2% (*n* = 37). The frequencies of the *NR3C1* and *GLCCI1* gene polymorphisms in our cohort were almost comparable to those reported in previous studies [18, 19].

**Table 1:** Patient, cord blood unit, and transplant characteristics

Characteristic	Value
Number of CBT	159
Age	
< 50 years	82 (51.6)
≥ 50 years	77 (48.4)
Sex	
Male	100 (62.9)
Female	59 (37.1)
HCT-CI	
0–2	135 (84.9)
≥ 3	24 (15.1)
Diagnosis	
AML	85 (53.5)
MDS	21 (13.2)
ALL	31 (19.5)
CML	7 (4.4)
CMML/MPN	6 (3.7)
NHL/ATL	4 (2.5)
CAEBV	3 (1.8)
Others	2 (1.2)
Refined disease risk index	
Low/intermediate/undetermined	74 (46.5)
High/very high	85 (53.5)
Conditioning regimen	
MAC	148 (93.1)
RIC	11 (6.9)
GVHD prophylaxis	
CSP + MTX	89 (56.0)
Others	70 (44.0)
Cryopreserved TNC dose	
< 2.5 × 10^7^/kg	67 (42.1)
≥ 2.5 × 10^7^/kg	92 (57.9)
Cryopreserved CD34⁺ cell dose	
< 1.0 × 10^5^/kg	73 (45.9)
≥ 1.0 × 10^5^/kg	86 (54.1)
HLA disparities^*^	
0–1	40 (25.2)
2	119 (74.8)
Sex incompatibility	
Female donor to male recipient	39 (24.5)
Others	120 (75.5)
Number of allogeneic HCT	
1	127 (80.0)
2	32 (20.0)
Recipient res33388 polymorphism	
TT	104 (65.8)
AT	50 (31.7)
AA	4 (2.5)
Recipient rs37972 polymorphism	
CC	59 (37.3)
TC	24 (15.2)
TT	75 (47.5)
Recipient rs37973 polymorphism	
GG	27 (17.1)
GA	83 (52.5)
AA	48 (30.4)
Donor res33388 polymorphism	
TT	92 (67.7)
AT	41 (30.2)
AA	3 (2.2)
Donor rs37972 polymorphism	
CC	47 (34.6)
TC	27 (19.9)
TT	62 (45.6)
Donor rs37973 polymorphism	
GG	35 (25.7)
GA	64 (47.1)
AA	37 (27.2)

CBT, cord blood transplantation; HCT-CI, Hematopoietic Cell Transplantation-Specific Comorbidity Index; AML, acute myeloid leukemia; MDS, myelodysplastic syndrome; ALL, acute lymphoblastic leukemia; CML, chronic myelogeneous leukemia; CMML, chronic myelomonocytic leukemia; MPN myeloproliferative neoplasm; NHL, non-Hodgkin’s lymphoma; ATL, adult T-cell leukemia; CAEBV, chronic active Epstein-Barr virus infection; MAC, myeloablative conditioning; RIC, reduced intensity conditioning; GVHD, graft-versus-host disease; CSP, cyclosporine; MTX, methotrexate; TNC, total nucleated cell; HLA, human leukocyte antigen; HCT, hematopoietic cell transplantation

^*^HLA disparities between the cord blood graft and the recipient were defined as a low-resolution for HLA-A, HLA-B, and HLA-DR in graft-versus-host direction

### Acute and chronic GVHD

**Fig. 1:**
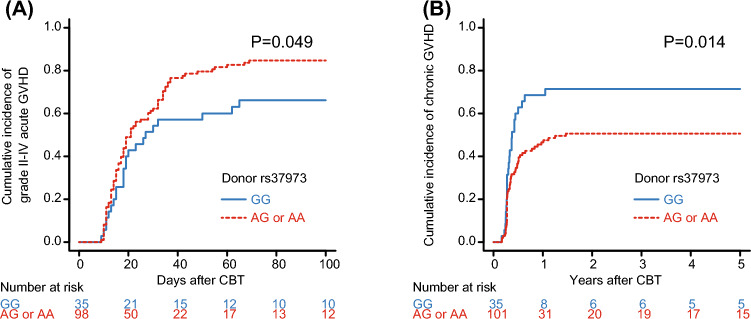
Unadjusted cumulative incidences of grade II-IV acute graft-versus-host disease (GVHD) (**A**) and chronic GVHD (**B**) following single-unit cord blood transplantation (CBT) according to donor polymorphism rs37973 in the *GLCCI1* gene

In the univariate analyses, the cumulative incidence of grades II to IV acute GVHD was significantly higher in *GLCCI1* rs37973 AG or AA donors compared to GG donors (84.7% vs. 66.2% at 100 days, *P* = 0.049) (Fig. 1A, Table 2). In contrast, the cumulative incidence of chronic GVHD was significantly higher in *GLCCI1* rs37973 GG donors compared to AG or AA donors (71.4% vs. 50.6% at 2 years, *P* = 0.014) (Fig. 1B, Table 2). The multivariate analyses showed that an rs37973 AG or AA donor had a significantly lower risk of chronic GVHD compared to a GG donor (hazard ratio [HR], 0.57; 95% confidence interval [CI], 0.35–0.93; *P* = 0.025), but the donor rs37973 polymorphism was associated with a marginally increased risk of grades II to IV acute GVHD (HR, 1.60; 95% CI, 0.95–2.68; *P* = 0.074) (Table 3). The rs33388, rs37972, and rs37973 polymorphisms of recipients and donors did not affect grades II, and grades III to IV acute GVHD, and moderate to severe chronic GVHD (Tables 2,3).

**Table 2:** Univariate analysis of acute and chronic graft-versus-host disease (GVHD) according to recipient and donor gene polymorphism of rs33388, rs37972, and rs37973

	Grades II acute GVHD		Grades II-IV acute GVHD		Grades III-IV acute GVHD		Chronic GVHD all grade		Chronic GVHD moderate/severe	
	% at 100 days (95%CI)	*P*	% at 100 days (95%CI)	*P*	% at 100 days (95%CI)	*P*	% at 2 years (95%CI)	*P*	% at 2 years (95%CI)	*P*
Recipient rs33388										
TT	56.0 (45.6–65.1)	0.530	77.0 (67.3–84.2)	0.876	21.0 (13.6–29.5)	0.524	54.9 (44.7–63.9)	0.243	20.2 (13.1–28.4)	0.221
AT or AA	62.6 (47.7–74.3)		79.9 (65.5–88.7)		17.0 (8.3–28.4)		48.3 (34.2–61.0)		13.0 (5.6–23.4)	
Recipient rs37972										
CC	55.2 (41.3–67.0)	0.610	79.3 (66.0–87.9)	0.456	24.1 (14.0–35.8)	0.231	50.8 (37.3–62.9)	0.970	22.0 (12.4–33.4)	0.262
TC or TT	60.1 (49.4–69.2)		77.0 (67.0–84.4)		16.9 (10.1–25.1)		53.6 (50.1–63.0)		15.2 (8.9–23.0)	
Recipient rs37973										
GG	70.4 (48.4–84.4)	0.146	77.8 (55.7–89.8)	0.909	7.4 (1.2–21.4)	0.077	63.0 (41.1–78.6)	0.408	18.7 (6.6–35.6)	0.910
AG or AA	55.6 (46.4–63.8)		77.9 (69.4–84.2)		22.3 (15.4–29.9)		50.5 (41.6–58.7)		17.6 (11.6–24.6)	
Donor rs33388										
TT	57.8 (46.8–67.3)	0.852	80.0 (70.0–87.0)	0.714	22.2 (14.3–31.3)	0.635	56.7 (45.8–66.2)	0.877	17.4 (10.4–25.8)	0.846
AT or AA	60.9 (44.3–74.0)		79.8 (63.4–89.5)		18.6 (8.6–31.5)		54.5 (38.5–68.1)		16.1 (7.0–28.5)	
Donor rs37972										
CC	59.8 (44.0–72.4)	0.860	77.0 (61.4–86.9)	0.241	17.0 (7.9–29.1)	0.406	53.2 (37.8–66.4)	0.868	23.4 (12.4–36.3)	0.131
TC or TT	58.1 (46.9–67.8)		81.4 (71.2–88.3)		23.3 (14.9–32.7)		57.5 (46.4–67.1)		13.5 (7.4–21.5)	
Donor rs37973										
GG	48.9 (31.2–64.4)	0.169	66.2 (47.3–79.7)	**0.049**	17.1 (6.8–31.4)	0.553	71.4 (52.6–83.8)	**0.014**	17.3 (6.9–31.6)	0.996
AG or AA	62.2 (51.8–71.1)		84.7 (75.7–90.6)		22.5 (14.8–31.2)		50.6 (40.4–60.0)		16.8 (10.3–24.8)	

CI, confidence interval

The *P* values in bold are statistically significant

**Table 3:** Multivariate analysis of acute and chronic graft-versus-host disease (GVHD) according to recipient and donor gene polymorphism of rs33388, rs37972, and rs37973

	Grades II acute GVHD		Grades II-IV acute GVHD		Grades III-IV acute GVHD		Chronic GVHD all grade		Chronic GVHD moderate/severe	
	Adjusted HR (95%CI)	*P*	Adjusted HR (95%CI)	*P*	Adjusted HR (95%CI)	*P*	Adjusted HR (95%CI)	*P*	Adjusted HR (95%CI)	*P*
Recipient rs33388										
TT	1.00		1.00		1.00		1.00		1.00	
AT or AA	1.14 (0.73–1.76)	0.560	0.96 (0.66–1.40)	0.860	0.73 (0.31–1.74)	0.490	0.76 (0.48–1.22)	0.270	0.62 (0.25–1.52)	0.300
Recipient rs37972										
CC	1.00		1.00		1.00		1.00		1.00	
TC or TT	1.07 (0.69–1.68)	0.740	0.86 (0.59–1.25)	0.450	0.56 (0.26–1.21)	0.140	0.97 (0.58–1.63)	0.930	0.68 (0.29–1.57)	0.370
Recipient rs37973										
GG	1.00		1.00		1.00		1.00		1.00	
AG or AA	0.73 (0.43–1.23)	0.240	1.06 (0.66–1.71)	0.780	3.46 (0.78–15.22)	0.100	0.89 (0.52–1.53)	0.690	0.98 (0.33–2.89)	0.990
Donor rs33388										
TT	1.00		1.00		1.00		1.00		1.00	
AT or AA	0.92 (0.55–1.53)	0.760	0.87 (0.57–1.31)	0.510	0.89 (0.37–2.14)	0.800	0.97 (0.58–1.63)	0.930	0.65 (0.19–2.18)	0.490
Donor rs37972										
CC	1.00		1.00		1.00		1.00		1.00	
TC or TT	1.07 (0.68–1.68)	0.770	1.35 (0.91–1.99)	0.130	1.51 (0.66–3.44)	0.320	1.01 (0.60–1.68)	0.960	0.38 (0.12–1.16)	0.090
Donor rs37973										
GG	1.00		1.00		1.00		1.00		1.00	
AG or AA	1.71 (0.94–3.12)	0.078	1.60 (0.95–2.68)	0.074	0.87 (0.31–2.44)	0.800	0.57 (0.35–0.93)	**0.025**	1.45 (0.41–5.01)	0.560

HR, hazard ratio; CI, confidence interval

The *P* values in bold are statistically significant

Among acute GVHD and PES/PIR, the rs33388, rs37972, and rs37973 polymorphisms of recipients and donors were not associated with organ-specific acute GVHD, such as skin, liver, or gut, and PES/PIR in univariate (data not shown) and multivariate analysis (Supplementary Table 1).

**Fig. 2:**
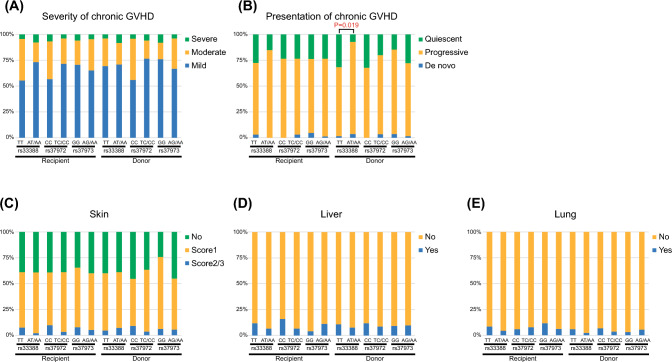
Severity (**A**), type of presentation (**B**), and organ involvement (**C-E**) of chronic graft-versus-host disease (GVHD) following single-unit cord blood transplantation according to recipient and donor gene polymorphism of rs33388, rs37972, and rs37973

Among chronic GVHD, the severity which is defined as the NIH classification, type of presentation, and organ involvement, were not associated with the rs33388, rs37972, and rs37973 polymorphisms of recipients and donors (Fig. 2A, C-E), except that the proportion of quiescent chronic GVHD was higher in *NR3C1* rs33388 TT donors compared to AT or AA donors (89.3% vs. 66.7%, *P* = 0.019) (Fig. 2B).

### BSI and CMV reactivation

Univariate analysis demonstrated that the cumulative incidence of CMV reactivation was significantly higher in *GLCCI1* rs37973 GG donors compared to AG or AA donors (85.7% vs. 65.3% at 100 days, *P* = 0.029) (Supplementary Fig. S1A). However, the rs33388, rs37972, and rs37973 polymorphisms of recipients and donors were not associated with the cumulative incidences of BSI in univariate analysis (data not shown). In the multivariate analysis, the rs33388, rs37972, and rs37973 polymorphisms of recipients and donors were not significantly associated with the probabilities of BSI and CMV reactivation (Supplementary Table S2).

### Relapse, NRM, and OS

None of the rs33388, rs37972, or rs37973 polymorphisms in recipients or donors significantly influenced relapse, NRM, or OS in either univariate or multivariate analysis (Supplementary Table S3, S4).

**Fig. 3:**
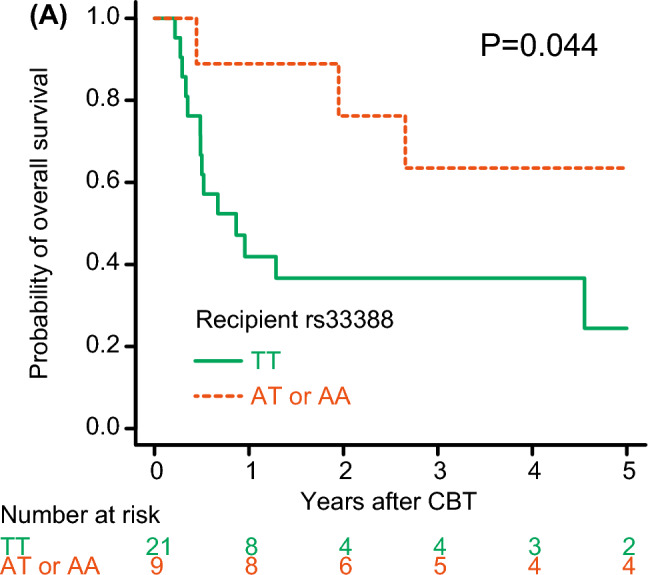
Unadjusted probability of overall survival following single-unit CBT among patients who developed grades III-IV acute GVHD treated with glucocorticoids according to recipient polymorphism rs33388 in the *NR3C1* gene (**A**)

We also examined the impact of these polymorphisms on OS among patients who developed grades III to IV acute GVHD treated with glucocorticoids. Among 30 evaluable patients, the probability of OS was significantly lower in *NR3C1* rs33388 TT recipients compared to AT or AA recipients in univariate analysis (24.4% vs. 63.5% at 5 years, *P* = 0.044) (Fig. 3).

### Subgroup analysis in acute leukemia

Because there was considerable heterogeneity in the underlying diseases among patients in our cohort, we conducted subgroup analyses limited to those with acute myeloid or lymphoblastic leukemia (*n* = 116). In univariate analysis, the cumulative incidence of chronic GVHD was also significantly higher in *GLCCI1* rs37973 GG donors compared to AG or AA donors (77.3% vs. 52.6% at 2 years, *P* = 0.005) (Supplementary Fig. S2A). Among patients who developed grades III to IV acute GVHD treated with glucocorticoids, the probability of OS was lower in *NR3C1* rs33388 TT recipients compared to AT or AA recipients, but this was a marginal difference (26.1% vs. 71.4% at 5 years, *P* = 0.072) (Supplementary Fig. S2B).

## Discussion

Our study indicated that the *GLCCI1* rs37973 GG donor had an increased risk of chronic GVHD compared to rs37973 AG or AA donors. In contrast, the *GLCCI1* rs37973 GG donor had a decreased risk of grades II to IV acute GVHD compared to rs37973 AG or AA donors in the univariate analysis, but this association was only marginally significant in the multivariate model. Although the risk for chronic GVHD went up with the grade of acute GVHD [20, 21], the donor rs37973 polymorphism had the opposite effect on both acute and chronic GVHD in our study.

Several studies investigated the association between recipient or donor gene polymorphism involving glucocorticoid responsiveness and GVHD in allogeneic HCT [9–11]. O’Meara, et al. reported that glucocorticoid receptor, *NR3C1*, rs41423247 polymorphism in the recipient was associated with responsiveness to glucocorticoid, but rs37973 in the recipient and donor was not associated with the development of acute and chronic GVHD after allogeneic HCT [9]. The A allele of rs33388 in the recipient was linked to a higher risk of developing grades II to IV acute GVHD after allogeneic HCT [11]. Consistent with clinical data, several murine studies have shown that deficiency of the glucocorticoid receptor in either donor or recipient compartments exacerbates GVHD [22, 23].

Li et al. showed that deletion of the glucocorticoid receptor in donor T cells caused fulminant acute GVHD with intestinal upregulation of inflammatory and metabolic genes, primarily driven by donor T cells rather than macrophages [22]. The mouse Glcci1 transcript is highly expressed in the thymus and testis, but it is weakly expressed in all other tissues [24]. As for *GLCCI1* polymorphism in the clinical setting, under the additive genetic model, rs37973 was associated with an increased odds of poor response to inhaled corticosteroid therapy in asthma [4], suggesting that the GG allele of rs37973 might be related to poor glucocorticoid responsiveness. The exact ways that the donor rs37973 polymorphism is linked to lower acute or higher chronic GVHD have not been fully explained, but we speculated that the differences in the responsiveness of donor lymphocytes to endogenous glucocorticoids may contribute to the distinct pathophysiology of acute and chronic GVHD. Thus, donor rs37973 GG homozygosity may confer glucocorticoid resistance in donor lymphocytes, contributing to higher chronic GVHD, whereas its limited impact during the early posttransplant period might result in lower acute GVHD.

We also demonstrated that the recipient rs33388 TT was significantly associated with lower OS among patients who developed grades III to IV acute GVHD treated with glucocorticoids. Norden et al. showed that the absence of the T allele rs33388 in recipients was associated with lower OS through increased relapse rates [11]. Given that the presence of the T allele rs33388 is associated with increased glucocorticoid responsiveness in patients with pemphigus vulgaris [3], our data means that patients with a glucocorticoid refractoriness phenotype in the recipient had better OS among patients who developed grades III to IV acute GVHD treated with glucocorticoids after CBT. Indeed, Baake et al. demonstrated that glucocorticoid receptor deficiency in recipient cells aggravated acute GVHD and mortality, with elevated serum IL-6 and IFN-γ levels in the murine model. Similar findings in mice lacking the receptor only in myeloid cells indicate its crucial role in suppressing the cytokine storm during acute GVHD [23]. The conflicting processes that explain the relationship between rs33388 TT recipients, who demonstrate favorable glucocorticoid responsiveness, and poorer OS have not been completely clarified, but glucocorticoid treatment in recipients carrying the rs33388 TT genotype may have excessively suppressed their immune defense against infections, which could contribute to lower OS in our study. These data also suggest that further study is needed to clarify the role of glucocorticoid receptor gene polymorphism and immune defense against infections in allogeneic HCT.

A major limitation in this study was a retrospective analysis as a single institution in Japan that included a relatively small number of patients. Furthermore, there might be the racial difference of gene polymorphism involving glucocorticoid responsiveness, which also influences the impacts of polymorphism on transplant outcomes. Therefore, the association between the gene polymorphism involving glucocorticoid responsiveness and CBT outcomes requires further investigation across diverse racial groups. Second, the gene polymorphism involving glucocorticoid responsiveness might differ their role in GVHD according to donor type. This might be partly due to the higher proportion of naïve phenotype in cord blood lymphocytes, which could contribute to the different responsiveness to glucocorticoids between adult donor HCT and CBT. Indeed, previous studies demonstrated a higher response rate to glucocorticoids for GVHD in CBT compared with adult donor HCT [25, 26]. Therefore, further investigation of gene polymorphism involving glucocorticoid responsiveness will elucidate their role in GVHD according to donor type. Third, the mechanisms by which the polymorphisms of *GLCCI1* and *NR3C1* modulate glucocorticoid responsiveness remain incompletely understood in the clinical setting. Indeed, innate immune activation and tissue injury—triggered by total body irradiation, high-intensity pre-transplant chemotherapy, or antibiotic exposure during the peri-engraftment phase—can modulate glucocorticoid receptor signaling and responsiveness.

In conclusion, we demonstrated that the absence of the A allele of donor *GLCCI1* rs37973 was associated with an increased risk of chronic GVHD in adults undergoing single-unit CBT. Among patients who developed grades III to IV acute GVHD treated with glucocorticoids, *NR3C1* rs33388 TT recipients had worse OS. Thus, the polymorphism of *GLCCI1* rs37973 and *NR3C1* rs33388 in recipients and donors might predict the risk of GVHD and survival after single-unit CBT. Further multicenter studies across diverse populations are warranted to clarify the role of glucocorticoid-related genetic variation in CBT outcomes.

## Supplementary Information

Below is the link to the electronic supplementary material.

Supplementary file1 (EPS 2342 KB)

Supplementary file2 (EPS 2739 KB)

Supplementary file3 (DOCX 21 KB)

Supplementary file4 (DOCX 19 KB)

Supplementary file5 (DOCX 20 KB)

Supplementary file6 (DOCX 19 KB)

## Data Availability

Data may be available from the corresponding author upon reasonable request.
