# Supplementary material for: Clinical impact of glucocorticoid responsiveness-related gene polymorphism on graft-versus-host disease and survival after single-unit cord blood transplantation
Source: Int J Hematol. 2025 Nov 20;123(3):412–20. doi: 10.1007/s12185-025-04112-y (PMC12967668; doi:10.1007/s12185-025-04112-y)
Supplement: Supplementary file 3 — Supplementary file3 (DOCX 21 KB) [file 12185_2025_4112_MOESM3_ESM.docx]

**Supplementary Table 1.** Multivariate analysis of organ-specific acute graft-versus-host disease (GVHD) and pre-engraftment syndrome (PES)/pre-engraftment immune reaction (PIR) according to recipient and donor gene polymorphism of rs33388, rs37972, and rs37973.

|  | Skin acute GVHD |  | Liver acute GVHD |  | Gut acute GVHD |  | PES/PIR |  |
| --- | --- | --- | --- | --- | --- | --- | --- | --- |
|  | Adjusted HR (95%CI) | P | Adjusted HR (95%CI) | P | Adjusted HR (95%CI) | P | Adjusted HR (95%CI) | P |
| Recipient rs33388 |  |  |  |  |  |  |  |  |
| TT | 1.00 |  | 1.00 |  | 1.00 |  | 1.00 |  |
| AT or AA | 0.88 (0.62-1.26) | 0.510 | 1.19 (0.37-3.78) | 0.770 | 1.05 (0.52-2.10) | 0.890 | 0.97 (0.63-1.49) | 0.910 |
| Recipient rs37972 |  |  |  |  |  |  |  |  |
| CC | 1.00 |  | 1.00 |  | 1.00 |  | 1.00 |  |
| TC or TT | 1.01 (0.71-1.42) | 0.950 | 0.45 (0.08-2.36) | 0.350 | 0.85 (0.45-1.62) | 0.640 | 0.68 (0.29-1.57) | 0.370 |
| Recipient rs37973 |  |  |  |  |  |  |  |  |
| GG | 1.00 |  | 1.00 |  | 1.00 |  | 1.00 |  |
| AG or AA | 1.18 (0.83-1.69) | 0.340 | 1.77 (0.19-16.41) | 0.610 | 1.59 (0.68-3.74) | 0.280 | 0.70 (0.44-1.13) | 0.150 |
| Donor rs33388 |  |  |  |  |  |  |  |  |
| TT | 1.00 |  | 1.00 |  | 1.00 |  | 1.00 |  |
| AT or AA | 0.84 (0.58-1.21) | 0.360 | 1.37 (0.46-4.09) | 0.570 | 0.96 (0.48-1.92) | 0.930 | 0.96 (0.62-1.51) | 0.890 |
| Donor rs37972 |  |  |  |  |  |  |  |  |
| CC | 1.00 |  | 1.00 |  | 1.00 |  | 1.00 |  |
| TC or TT | 1.05 (0.74-1.48) | 0.770 | 0.36 (0.11-1.20) | 0.097 | 1.48 (0.74-2.93) | 0.260 | 1.29 (0.81-2.06) | 0.280 |
| Donor rs37973 |  |  |  |  |  |  |  |  |
| GG | 1.00 |  | 1.00 |  | 1.00 |  | 1.00 |  |
| AG or AA | 0.91 (0.66-1.26) | 0.610 | 6.17 (0.81-46.70) | 0.078 | 1.14 (0.50-2.58) | 0.750 | 1.08 (0.65-1.80) | 0.740 |

HR, hazard ratio; CI, confidence interval.
