# Supplementary material for: Clinical impact of glucocorticoid responsiveness-related gene polymorphism on graft-versus-host disease and survival after single-unit cord blood transplantation
Source: Int J Hematol. 2025 Nov 20;123(3):412–20. doi: 10.1007/s12185-025-04112-y (PMC12967668; doi:10.1007/s12185-025-04112-y)
Supplement: Supplementary file 4 — Supplementary file4 (DOCX 19 KB) [file 12185_2025_4112_MOESM4_ESM.docx]

**Supplementary Table 2.** Multivariate analysis of bacterial bloodstream infection (BSI) and cytomegalovirus (CMV) reactivation according to recipient and donor gene polymorphism of rs33388, rs37972, and rs37973.

|  | Bacterial BSI |  | CMV reactivation |  |
| --- | --- | --- | --- | --- |
|  | Adjusted HR (95%CI) | P | Adjusted HR (95%CI) | P |
| Recipient rs33388 |  |  |  |  |
| TT | 1.00 |  | 1.00 |  |
| AT or AA | 1.15 (0.58-2.26) | 0.680 | 0.69 (0.46-1.04) | 0.082 |
| Recipient rs37972 |  |  |  |  |
| CC | 1.00 |  | 1.00 |  |
| TC or TT | 0.58 (0.29-1.13) | 0.110 | 1.19 (0.80-1.76) | 0.380 |
| Recipient rs37973 |  |  |  |  |
| GG | 1.00 |  | 1.00 |  |
| AG or AA | 2.64 (0.79-8.78) | 0.110 | 0.66 (0.42-1.05) | 0.084 |
| Donor rs33388 |  |  |  |  |
| TT | 1.00 |  | 1.00 |  |
| AT or AA | 1.20 (0.54-2.64) | 0.650 | 1.12 (0.74-1.71) | 0.580 |
| Donor rs37972 |  |  |  |  |
| CC | 1.00 |  | 1.00 |  |
| TC or TT | 0.83 (0.39-1.76) | 0.630 | 1.11 (0.72-1.69) | 0.620 |
| Donor rs37973 |  |  |  |  |
| GG | 1.00 |  | 1.00 |  |
| AG or AA | 1.33 (0.60-2.97) | 0.470 | 0.77 (0.49-1.23) | 0.290 |

HR, hazard ratio; CI, confidence interval.
