# Supplementary material for: Clinical impact of glucocorticoid responsiveness-related gene polymorphism on graft-versus-host disease and survival after single-unit cord blood transplantation
Source: Int J Hematol. 2025 Nov 20;123(3):412–20. doi: 10.1007/s12185-025-04112-y (PMC12967668; doi:10.1007/s12185-025-04112-y)
Supplement: Supplementary file 5 — Supplementary file5 (DOCX 20 KB) [file 12185_2025_4112_MOESM5_ESM.docx]

**Supplementary Table 3.** Univariate analysis of relapse, non-relapse mortality (NRM), and overall survival (OS) according to recipient and donor gene polymorphism of rs33388, rs37972, and rs37973.

|  | Relapse |  | NRM |  | OS |  |
| --- | --- | --- | --- | --- | --- | --- |
|  | % at 5 years (95%CI) | P | % at 5 years (95%CI) | P | % at 5 years (95%CI) | P |
| Recipient rs33388 |  |  |  |  |  |  |
| TT | 23.9 (15.7-33.1) | 0.156 | 17.6 (10.6-26.0) | 0.383 | 62.5 (51.3-71.8) | 0.856 |
| AT or AA | 13.1 (5.7-23.6) |  | 24.9 (14.0-37.4) |  | 61.8 (47.1-73.5) |  |
| Recipient rs37972 |  |  |  |  |  |  |
| CC | 21.8 (11.8-33.8) | 0.563 | 21.2 (11.6-32.8) | 0.962 | 63.1 (48.4-74.6) | 0.805 |
| TC or TT | 18.7 (11.6-27.2) |  | 19.7 (12.1-28.8) |  | 61.8 (50.5-71.2) |  |
| Recipient rs37973 |  |  |  |  |  |  |
| GG | 19.7 (6.9-37.3) | 0.787 | 13.0 (3.1-30.4) | 0.320 | 71.1 (48.3-85.2) | 0.285 |
| AG or AA | 20.2 (13.5-27.8) |  | 21.6 (14.7-29.3) |  | 60.4 (50.8-68.8) |  |
| Donor rs33388 |  |  |  |  |  |  |
| TT | 19.1 (11.3-28.4) | 0.152 | 18.6 (11.1-27.6) | 0.509 | 65.9 (54.4-75.1) | 0.705 |
| AT or AA | 9.8 (3.0-21.3) |  | 19.7 (9.0-33.6) |  | 71.9 (54.4-83.6) |  |
| Donor rs37972 |  |  |  |  |  |  |
| CC | 21.0 (10.1-34.7) | 0.357 | 18.4 (8.3-31.5) | 0.975 | 65.2 (48.5-77.7) | 0.574 |
| TC or TT | 13.0 (6.9-21.3) |  | 19.4 (11.5-28.8) |  | 68.8 (57.0-78.0) |  |
| Donor rs37973 |  |  |  |  |  |  |
| GG | 11.8 (3.6-25.3) | 0.469 | 12.1 (3.7-25.7) | 0.357 | 74.7 (55.2-86.7) | 0.318 |
| AG or AA | 17.5 (10.3-26.2) |  | 21.5 (13.6-30.6) |  | 65.1 (53.9-74.2) |  |

CI, confidence interval.
