# Supplementary material for: Clinical impact of glucocorticoid responsiveness-related gene polymorphism on graft-versus-host disease and survival after single-unit cord blood transplantation
Source: Int J Hematol. 2025 Nov 20;123(3):412–20. doi: 10.1007/s12185-025-04112-y (PMC12967668; doi:10.1007/s12185-025-04112-y)
Supplement: Supplementary file 6 — Supplementary file6 (DOCX 19 KB) [file 12185_2025_4112_MOESM6_ESM.docx]

**Supplementary Table 4.** Multivariate analysis of relapse, non-relapse mortality (NRM), and overall survival (OS) according to recipient and donor gene polymorphism of rs33388, rs37972, and rs37973.

|  | Relapse |  | NRM |  | OS |  |
| --- | --- | --- | --- | --- | --- | --- |
|  | Adjusted HR (95%CI) | P | Adjusted HR (95%CI) | P | Adjusted HR (95%CI) | P |
| Recipient rs33388 |  |  |  |  |  |  |
| TT | 1.00 |  | 1.00 |  | 1.00 |  |
| AT or AA | 0.54 (0.20-1.44) | 0.220 | 1.60 (0.73-3.54) | 0.240 | 1.22 (0.68-2.20) | 0.493 |
| Recipient rs37972 |  |  |  |  |  |  |
| CC | 1.00 |  | 1.00 |  | 1.00 |  |
| TC or TT | 0.78 (0.37-1.65) | 0.530 | 0.83 (0.38-1.80) | 0.650 | 0.98 (0.56-1.73) | 0.961 |
| Recipient rs37973 |  |  |  |  |  |  |
| GG | 1.00 |  | 1.00 |  | 1.00 |  |
| AG or AA | 1.20 (0.46-3.12) | 0.700 | 1.63 (0.50-5.32) | 0.410 | 1.34 (0.59-3.03) | 0.478 |
| Donor rs33388 |  |  |  |  |  |  |
| TT | 1.00 |  | 1.00 |  | 1.00 |  |
| AT or AA | 0.74 (0.27-2.06) | 0.580 | 1.01 (0.38-2.70) | 0.970 | 0.89 (0.42-1.88) | 0.774 |
| Donor rs37972 |  |  |  |  |  |  |
| CC | 1.00 |  | 1.00 |  | 1.00 |  |
| TC or TT | 0.89 (0.37-2.16) | 0.810 | 0.85 (0.36-1.98) | 0.710 | 0.81 (0.41-1.59) | 0.549 |
| Donor rs37973 |  |  |  |  |  |  |
| GG | 1.00 |  | 1.00 |  | 1.00 |  |
| AG or AA | 0.77 (0.21-2.79) | 0.700 | 2.30 (0.84-6.26) | 0.100 | 1.20 (0.52-2.77) | 0.663 |

HR, hazard ratio; CI, confidence interval.
